# Diabetes in Appalachia: providers’ perspectives

**DOI:** 10.1017/S1463423620000134

**Published:** 2020-05-14

**Authors:** Elizabeth A. Beverly, Marilyn D. Ritholz, Karie Cook, Lesli K. Johnson, Anirudh Ruhil, Rashmi P. Singh, Darlene E. Berryman

**Affiliations:** 1 Department of Family Medicine, Ohio University Heritage College of Osteopathic Medicine, Athens, OH 45701, USA; 2 The Diabetes Institute, Ohio University, Athens, OH 45701, USA; 3 Department of Behavioral Health, Joslin Diabetes Center, Boston, MA 02215, USA; 4 Department of Psychiatry, Harvard Medical School, Boston, MA 02215, USA; 5 Voinovich School of Leadership and Public Affairs, Ohio University, Athens, OH 45701, USA; 6 Department of Biomedical Sciences, Ohio University Heritage College of Osteopathic Medicine, Athens, OH 45701, USA

**Keywords:** diabetes, Appalachia, rural healthcare, barriers to healthcare, social determinants of health, qualitative methods

## Abstract

**Background:**

Southeastern Appalachian Ohio has more than double the national average of diabetes and a critical shortage of healthcare providers. Paradoxically, there is limited research focused on primary care providers’ experiences treating people with diabetes in this region. This study explored providers’ perceived barriers to and facilitators for treating patients with diabetes in southeastern Appalachian Ohio.

**Methods:**

We conducted in-depth interviews with healthcare providers who treat people with diabetes in rural southeastern Ohio. Interviews were transcribed, coded, and analyzed via content and thematic analyses using NVivo 12 software (QSR International, Chadstone, VIC, Australia).

**Results:**

Qualitative analysis revealed four themes: (1) patients’ diabetes fatalism and helplessness: providers recounted story after story of patients believing that their diabetes was inevitable and that they were helpless to prevent or delay diabetes complications. (2) Comorbid psychosocial issues: providers described high rates of depression, anxiety, incest, abuse, and post-traumatic stress disorder among people with diabetes in this region. (3) Inter-connected social determinants interfering with diabetes care: providers identified major barriers including lack of access to providers, lack of access to transportation, food insecurity, housing insecurity, and financial insecurity. (4) Providers’ cultural understanding and recommendations: providers emphasized the importance of understanding of the values central to Appalachian culture and gave culturally attuned clinical suggestions for how to use these values when working with this population.

**Conclusions:**

Evidence-based interventions tailored to Appalachian culture and training designed to increase the cultural competency and cultural humility of primary care providers may be effective approaches to reduce barriers to diabetes care in Appalachian Ohio.

Appalachia is a 205 000-square-mile region that encompasses 420 counties in 13 states from Mississippi to New York (The Appalachian Region, 2019a). The Appalachian Mountains are the major geographic feature of this region (The Appalachian Region, 2019a). In the state of Ohio, the Appalachian region encompasses 32 counties, of which 16 are designated as economically ‘at-risk’ or ‘distressed’ (The Appalachian Region, 2019b). The designation of economically ‘distressed’ is assigned for a county with a per capita income of 80% or less of the national average and/or an unemployment rate at least 1% greater than the national average unemployment rate for a 24-month period (The Appalachian Region, 1965). Here, 17.2% of the population lives below the poverty line as compared to 14.4% for the rest of the state (The Appalachian Region, 2019e) and 11.8% for the United States (US) (Smemega *et al.*, 2019). People are more likely to be unemployed (5.0% Appalachian Ohio versus 4.4% US), have lower educational achievement (e.g., percent with completed bachelor’s degree or more: 27.2% Appalachian Ohio versus 30.9% US), and limited access to transportation (Pollard and Jacobsen, 2019b; The Appalachian Region, 2020a). Moreover, Appalachian Ohio’s population has declined over the past 7 years with an approximate 2.9% decrease in adults under the age of 65 years (Pollard and Jacobsen, 2019a). Despite this decrease, the population has become more racially and ethnically diverse, though the percentage of this increase is smaller than the US overall (2.2% Appalachian Ohio versus 3.1% US) (Pollard and Jacobsen, 2019a). Consequently, these social determinants of health and secular trends are contributing to health disparities observed in people living in Appalachian Ohio (The Appalachian Region, 2017a).

One health disparity disproportionately affecting people in Appalachian Ohio is diabetes (2017a). An alarming 19.9% of adults in southeastern Ohio have diabetes (Ruhil *et al.*, 2017), which is more than double the national average of 9.4% (The Appalachian Region, 2017b). In this region, people with diabetes are more likely to have a delayed diabetes diagnosis, limited access to healthcare, lower health literacy, and lower empowerment (Zaugg *et al.*, 2014; de Groot *et al.*, 2007). For these reasons, people with diabetes are more likely to suffer from macrovascular and microvascular complications, lower limb amputation, and depression (de Groot *et al.*, 2007; Schwartz *et al.*, 2009). Despite the high rates of diabetes in the region, the impact of diabetes in rural Appalachian Ohio is understudied.

To date, limited research has focused on primary care providers treating diabetes in Appalachian Ohio. Specifically in southeastern Ohio, all seven of the counties are designated as health professional shortage areas for dental and mental healthcare; and, four counties are designated as health professional shortage areas for primary care (The Appalachian Region, 2019c; 2019d). High rates of diabetes coupled with shortages in providers create additional challenges to treating people with diabetes in this region. Thus, the purpose of this study was to increase our understanding of healthcare providers’ perceived barriers to and solutions for treating people with diabetes in this region. The information generated from this study will help develop relevant and practical approaches to diabetes treatment in rural Appalachia.

## Research design and method

### Research design

We conducted in-depth face-to-face and telephone interviews with healthcare providers who treat people with diabetes in southeastern Ohio using focused ethnographic methods. Focused ethnography is a means to access beliefs and practices in the context in which they occur, thereby improving our understanding of factors surrounding health and disease (Morse and Field 1996). As in other forms of qualitative research, the data collected are rich in their descriptions of beliefs, experiences, and behaviors of a population. For the purposes of this study, Appalachian Ohio was the sociocultural context of the participants. In addition to the sociocultural context, the participants all provided diabetes care to patients in the region. Thus, they shared the following beliefs and practices: diagnosis and treatment of diabetes, diabetes education practices (e.g., taking medication, monitoring blood glucose levels), diabetes beliefs (e.g., hypoglycemia is dangerous), and diabetes language (e.g., Hemoglobin A_1c_, retinopathy).

### Sample

We employed maximum variation sampling, a form of purposive sampling (Morse and Field, 1995b), to recruit (1) English-speaking healthcare providers with at least 5% of their practice consisting of patients with diabetes in order to ensure experience with treating diabetes; (2) providers practicing for a minimum of 5 years in rural southeastern Ohio (Athens, Hocking, Meigs, Morgan, Perry, Washington, Vinton Counties). Providers were recruited via emails, telephone calls, and word of mouth, and potential participants were screened by telephone for eligibility and general sociodemographic information. The authors assert that all procedures contributing to this work comply with the ethical standards of the relevant national and institutional guidelines on human experimentation by The Ohio University Office of Research Compliance (Institutional Review Board #16-E-342) and with the Helsinki Declaration of 1975, as revised in 2008. Written (face-to-face interviews) and verbal (telephone interviews) informed consent was obtained from all participants.

### Data collection

An experienced qualitative researcher conducted all interviews (E.A.B.), asking participants’ broad, open-ended questions about barriers unique to treating diabetes in Appalachia. Further, participants were asked how Appalachian culture might influence diabetes self-care behaviors and beliefs toward diabetes. The interviewer used directive probes to elicit additional information and clarify questions (see Table 1). In-person interviews were conducted at medical offices, conference rooms, and university offices; the remaining interviews were conducted via the telephone. Interviews lasted 30–90 min. Data were collected until saturation was reached; that is until no new information was generated from the interviews (Morse and Field, 1995a). All interviews were digitally audio-recorded and transcribed verbatim. The researchers performed quality checks of the transcribed files while listening to the interview recordings to validate the transcriptions. Participants’ names and identifiers were removed to protect provider confidentiality.

**Table 1.** Interview guide

Questions
1. Describe your role in the treatment of diabetes?
Probe: What is your role on the healthcare team?
Probe: How long have you been treating people with diabetes in this community?
2. In your own words, how would you describe the culture of Appalachia?
Probe: How might the culture of Appalachia affect diabetes rates in the region?
3. In general, how is diabetes affecting people in Appalachian Ohio?
Probe: How is diabetes viewed in Appalachian Ohio? What do people say about it?
4. What are the biggest barriers to diabetes care in rural Appalachia?
Probe: What are the biggest professional barriers that you experience?
Probe: What are the biggest patient barriers?
Probe: What are the biggest healthcare system barriers?
5. How do you try to address these barriers when treating people with diabetes?
Probe: How do you best collaborate with people with diabetes in the treatment relationship?
6. What support and resources do you need to take better care of people with diabetes in this region?
7. What can the healthcare community do to help you take care of people with diabetes?
8. Is there anything else about diabetes in this region that you want to share?

### Qualitative analysis

The multidisciplinary research team, consisting of behavioral diabetes and qualitative methodologist, two clinical psychologists, a nurse, a research methodologist, a physician, and a registered dietitian, used standard qualitative methods to analyze the data. Specifically, two members of the research team (E.A.B., R.P.S.) performed content analysis by independently marking and categorizing key words, phrases, and texts to identify themes (Pope and Mays, 2000; Krippendorf, 2004; Neuendorf, 2002). They met to code the data; discrepancies were reviewed, discussed, and resolved through consensus to establish inter-coder reliability (Neuendorf, 2002). The Cohen’s kappa coefficient between the two coders was 0.953, indicating almost perfect agreement (Cohen, 1968; Landis and Koch, 1977). No negative or deviant cases were excluded from the analysis (Lincoln and Guba, 1985). After the transcripts were coded and reviewed, one member of the research team (E.A.B.) entered the coded transcripts in NVivo 12 software (QSR International, Chadstone, VIC, Australia) to organize the coded data. The research team selected themes that characterized the participants’ perceived barriers and facilitators to diabetes treatment that occurred multiple times, both within and across transcripts.

### Rigor

To support credibility (validity), we employed investigator triangulation with experts from different disciplines. Utilizing multiple analyst triangulation to review our findings provided a check on potential selective perceptions as well as a means to identify cognitive biases in the analysis (Miles and Huberman, 1994). Further, four participants reviewed the findings to achieve participant corroboration and establish validity of the accounts (Denzin, 1978). Transferability (external validity) was supported via rich descriptions of the culture and verbatim quotations included in the data (Geertz, 1973). To support dependability (reliability), a researcher not involved with the study conducted an inquiry audit to examine the research process and evaluate whether or not the findings and conclusions were supported by the data (Lincoln and Guba, 1985).

## Results

Forty-two providers from diverse training backgrounds participated in 23 in-person interviews and 19 telephone interviews (see Table 2). The average age of participants was 45.1 ± 12.0 years. More than half (59.5, *n* = 25) identified as primary care providers, 66.7% (*n* = 28) identified as female, and 95.2% (*n* = 40) self-reported as white. The mean number of years in clinical practice was 17.7 ± 11.5, with an estimated diabetes caseload of 46.8 ± 29.6% (see Table 2). Transcript identifiers are used with quotations indicating participant number and provider type. The following themes emerged:

**Table 2.** Participants’ demographic and practice characteristics (*n* = 42)

Variable	*n* (%)
Age (years)	45.1 ± 12.0
Sex	
Female	28 (66.7)
Male	14 (33.3)
Ethnicity	
Hispanic/Latino	0 (0)
Race	
American Indian or Alaska Native	1 (2.4)
Asian	1 (2.4)
White	40 (95.2)
Provider type	
Physician	
Primary care	11 (26.2)
Endocrinology or diabetology	3 (7.1)
Surgeon	1 (2.4)
Nurse	9 (21.4)
Nurse practitioner	5 (11.9)
Pharmacist	3 (7.1)
Certified diabetes educator	3 (7.1)
Clinical psychologist	3 (7.1)
Exercise physiologist	2 (4.8)
Registered dietitian	1 (2.4)
Emergency medical technician	1 (2.4)
Practicing county	
All seven southeastern Ohio counties	5 (11.9)
Athens, OH	23 (54.8)
Hocking, OH	2 (4.8)
Meigs, OH	4 (9.5)
Morgan, OH	2 (4.8)
Perry, OH	3 (7.1)
Washington, OH	2 (4.8)
Vinton, OH	1 (2.4)
Years in practice (years)	17.7 ± 11.5
Employment status	
Part-time	2 (4.8)
Full-time	40 (95.2)
Estimated percentage of patients with diabetes (%)	46.8 ± 29.6

### Theme 1: Diabetes fatalism and helplessness

Nearly, all of the providers (*n* = 37 out of 42; no differences observed between in-person and telephone interviews, *χ* ^2^ = 4.468, *P* = 0.107) reported patients’ perceptions of diabetes fatalism as a major barrier to treating diabetes in the region. Providers recounted story after story of patients believing that their diabetes was inevitable because everyone in their family had it and that they were helpless to prevent or delay diabetes complications:

The fatalistic attitude is because they see so many people around them, and everybody they talk to today either has diabetes or has a family member or neighbor or knows somebody with diabetes, so it’s a given. Everybody’s got diabetes, that’s what you hear today. (ID 24, Certified Diabetes Educator)

Further, providers described patients feeling resigned to a life with diabetes where they felt powerless to affect the onslaught of inevitable complications and eventual death. Providers commented that these fatalistic attitudes made treating diabetes more challenging because their patients felt less hopeful about the disease than they did:

I think diabetes is looked at as something that’s just going to happen. Every one of my family members has it. I’m going to get it eventually. I’ll just deal with it when I get to it… They’ve watched their family members die of complications of diabetes. They just accept ‘That’s my fate.’ (ID 14, Primary Care Physician)

I feel like I’m pushing the 5,000-ton boulder all the time in the sense that it could be better, that you can change this. I do not feel like that message is out there. They are not destined to have diabetes. They could make lifestyle changes to improve their health and either have very mild or well-controlled diabetes or reverse it completely. That is probably my hugest barrier because I feel more hopeful for them than they do. (ID 43, Primary Care Physician)

In the providers’ words, patients did not know or understand that they could perform self-care behaviors to prevent or delay the onset of diabetes or diabetes complications. Further, they explained that even if patients did understand that diabetes was associated with self-care behaviors, many could not afford to purchase healthier foods or access safe spaces to exercise:

I think that they see it as a problem, but they have sort of a fatalistic approach that, ‘Well, there’s not too much I can do about it. You know, I can’t afford to eat the right foods. My hills are uneven, so I can’t go out and walk for exercise.’ (ID 2, Primary Care Physician)

### Theme 2: Comorbid psychosocial issues

Providers (*n* = 32 out of 42; no differences observed between in-person and telephone interviews, *χ* ^2^ = 3.521, *P* = 0.061) reported high rates of comorbid mental illness in the region. They understood that psychosocial issues affected a larger proportion of people with diabetes compared to the general population. Specifically, they noted high rates of depression and anxiety in people with diabetes:

A lot of my patients have mental health issues; [in] my diabetes population probably about 50 percent of them do. Depression and anxiety, I just see that through and through, over and over, everyday. (ID 7, Nurse Practitioner)

Providers also talked about how treating diabetes with comorbid psychosocial issues was difficult given that southeastern Ohio was a health professional shortage area for mental health providers. With a lack of mental health professionals, providers could not refer their patients for proper treatment. Providers observed that patients with untreated mental health issues were less likely to engage in self-care behaviors and more likely to have blood glucose levels above target:

I think there’s a huge, huge comorbid crossover between mental illness – depression and anxiety – and diabetes care. That’s really associated at least with some of the non-compliance issues I’ve mentioned before. I don’t think it’s a secret that southeast Ohio is very understaffed as far as behavioral health professionals. What I’ve seen is those patients that have underlying mental health issues are more likely to end up hospitalized due to very erratic sugar. (ID 17, Primary Care Physician)

Several providers also discussed the pervasiveness of incest, abuse, and post-traumatic stress disorder (PTSD) in the region. Treating these issues appeared to induce a sense of helplessness and to take an emotional toll on these providers as they described their patients’ traumatic experiences. They spoke about the difficult task of trying to treat patients with diabetes who had experienced these horrific traumas:

There was one day when I first started here, and I was scheduled eight patients in the afternoon. They were all new to me. Five of them had been sexually abused, and one of them was a man who had been abused by his father. Another woman, who I think because of her sexual abuse, now has serious psychological problems. She became schizophrenic. Has had multiple suicide attempts, IV drug abuse, endocarditis. And there is no pill that fixes these things. There is no pill that fixes it. (ID 15, Primary Care Physician)

### Theme 3: Inter-connected social determinants interfering with diabetes care

All 42 providers stressed how high-risk social determinants of health interfered with their ability to treat diabetes effectively. Major barriers included lack of access to providers, lack of access to transportation, food insecurity, housing insecurity, and financial insecurity. All of the providers agreed that one social determinant of health often contributed to another social determinant of health. For example, when describing access to providers, transportation barriers were mentioned simultaneously:

We have three clinics in Meigs County. We have no hospitals. If they are in need of any long-term care, you have to travel at least 50 miles roundtrip to get to a facility where you get a lot of testing, and maybe education, and things like that. A lot of people say I can’t get there, so I’m not going to do anything about it… Transportation is one of the biggest barriers here. (ID 42, Nurse)

Similarly, when discussing transportation barriers, providers referred to problems with food insecurity. Providers talked about limited options for food and the high prevalence of food deserts in southeastern Ohio:

I think there are limited options for food. So driving the ambulance around the county, I can see that there’s food deserts, where there is no grocery store. The nearest grocery store is 40 minutes away. There are a lot of gas stations where people will get their meals from. There’s a lot of fast food options in Meigs County. (ID 10, Emergency Medical Technician)

Providers identified housing insecurity as a significant issue complicating diabetes management. As providers explained, if their patients are trying to find a place to stay, they most likely are trying to find food and employment. Therefore, everything is a barrier for their diabetes. They have to prioritize where they are going to sleep ahead of engaging in diabetes self-care behaviors:

Housing is another thing. I mean if you’ve got a homeless person they really have a hard time taking care of themselves. Their self-management is going to be really poor because they’re more worried about not having shelter, not having food, that kind of thing. The [low-income] housing is almost nonexistent here. (ID 29, Nurse)

Finally, providers cited the cost of diabetes supplies, in particular diabetes medications, as insurmountable. Even with state pharmaceutical assistance programs, the majority of these providers’ patients could not afford their diabetes medications:

Affordable medications, that’s a huge barrier. There’s patient assistance programs, but not everyone qualifies. And then if they don’t qualify, they still can’t afford their insulins, and I think that is a huge barrier. Because they need their insulin to survive, and they still can’t afford it. (ID 26, Nurse)

### Theme 4: Providers’ cultural understanding and recommendations

One-third of the providers (*n* = 14 out of 42) demonstrated a clear understanding of the values central to Appalachian culture: kinship, loyalty, generosity, and caregiving (Russ, 2010; Coyne *et al.*, 2006). Appalachian culture is family centered, including extended kin (e.g., neighbors, church members). Every member of the family is expected to contribute to the community, which helps to develop a strong sense of trust among community members. Providers explained the importance of building that sense of trust with their patients and how they like being recognized as part of the community:

In Appalachian culture, everyone is very, very close. Everyone knows everybody else’s business…I actually like that where I go to places, people know who I am. They’re very trusting. You have to gain their trust first. (ID 11, Pharmacist)

Importantly, these providers recognized that trust was crucial in the delivery of primary care to patients in Appalachia. Trust facilitated their patients’ healthcare-seeking behaviors, open dialog, and engagement in self-care. On the other hand, providers also understood that trust had to be earned by showing respect to the community and its cultural traditions. Providers suggested relationship-building behaviors, including practicing nonjudgmental communication and participating in meaningful activities of the community, as strategies to help build trust with their patients:

Appalachian culture, trust is a huge issue with this culture, establishing trust. I think I have a real good opportunity to develop trust with people, but it takes a while to develop that trust. I think as a provider the number one thing you have to do is be nonjudgmental of their life circumstances. You have to be willing to help them go through whatever barriers they need to. (ID 29, Nurse)

Show them that you really care. When their mom dies, go down to viewing hours and say, ‘I heard about your mom dying. I thought I’d come down and pay my respects’ and suddenly they may start listening to you when they think that you really care about what matters to them. It’s meaningful to the people that love them. So I go, and I pay my respects, and I talk to the family and say ‘You know, I really liked Joe’ or ‘I really liked Mary. She was a lovely lady. She always treated me really well.’ It’s sincere…It’s part of the culture to have a family reunion when someone dies. (ID 14, Primary Care Physician)

Finally, providers recognized that Appalachians take great pride in helping others in their community. The people of Appalachia are very independent and hardworking, so it may be difficult for them to accept assistance from others:

People don’t want to ask for handouts in Appalachia either. They want to be able to help other people; they want to be able to give, but they don’t want to ever have to ask for help or ask for handouts, and that’s part of the culture. I think it’s really hard for them to feel that they need somebody else, or need to depend on somebody. (ID 24, Certified Diabetes Educator)

Therefore, finding a way to involve community members in diabetes care may be one solution to increase patients’ engagement in self-care and reduce barriers to diabetes treatment:

I think the best thing that could happen for diabetes care is that the diabetes people who are really dedicated to it find a way to get out of that facility, get buy-in into a separate place that is within the community that people go to where they don’t have to go to the hospital, but they can come in and they’re treated by other Appalachians, maybe their cousin or their aunt or somebody, and they say ‘Okay, this is for us and by us. We manage it in a way that makes sense for us.’ And that the people researching them really understand and take the time to understand the culture of Appalachia. (ID 14, Primary Care Physician)

## Discussion

This qualitative study explored providers’ perspectives for treating diabetes in southeastern Appalachian Ohio. All of the providers emphasized the frequency and seriousness of social determinants of health and how they negatively affected diabetes management. Further complicating efforts to treat diabetes were patients’ widespread fatalistic attitudes and sense of helplessness toward diabetes. The majority of providers noted that patients felt predestined to develop diabetes because everyone in their family had it. They explained that their patients did not know or understand that they could perform behaviors to reduce the risk of developing diabetes or to prevent or delay diabetes complications. High rates of comorbid psychosocial issues, particularly depression, anxiety, and PTSD posed additional challenges and feelings of helplessness for both patients and providers. This region is a health professional shortage area for mental health providers; therefore, many patients did not receive the care that they needed. Further, individuals with untreated psychosocial issues were less likely to engage in self-care behaviors and have high blood glucose levels. To address the barriers identified by the providers, many stressed the importance of understanding Appalachian culture. These providers recognized that earning the trust of Appalachians and showing loyalty to the community were essential to developing an effective patient–provider relationship.

The clinical implications of these findings are of utmost importance considering the helplessness that both patients and providers appeared to feel when facing diabetes and its treatment. The construct of the ‘Invisible Outsider’ complements our providers’ cultural understanding and recommendations to address the perceived barriers to diabetes treatment in this study (Protivnak *et al.*, 2017). This concept described in Protivnak *et al.*’s (2017) article on psychological counseling of people from Appalachia highlights the importance of forming a working alliance with patients from Appalachia and using a multicultural framework. Forming a working alliance in diabetes treatment necessitates that providers establish a partnership with patients and that they are aware not only of patients’ beliefs and behaviors but also of their own responses to evidence of medical non-adherence (Ritholz, 2009). In a working alliance, patients and providers agree on goals, the tasks needed to accomplish those goals, and feel and demonstrate mutual respect and trust (Bordin, 1979). Further, the construct of ‘Invisible Outsiders’ also may allow providers to view Appalachia as a unique culture to explore with an anthropologically inquisitive frame of mind (Protivnak *et al.*, 2017). Consequently, if healthcare providers want to work with this population, they need to spend time on researching, understanding, and addressing the unique ways people with diabetes in Appalachia think about health and illness, psychosocial concerns, and patient and provider roles in the pursuit of treatment (Tseng *et al.*, 2013). For example, people from Appalachia are more likely to somatize compared to the general population, which can lead to self-medication and substance abuse (Keefe *et al.*, 2005; Russ, 2010). Providers’ understanding of the relationship between patients’ somatization and their overreliance on substances may assist with the forming of an effective working alliance. Finally, providers need to consider how patients conceptualize and implement self-care practices as well as understand and incorporate their folk and complementary medicines (Tseng *et al.*, 2013; Cavender, 2006). Thus, training primary care providers in cultural competency and cultural humility may be an important approach to reduce barriers to diabetes care in Appalachian Ohio.

A long history of isolation and outside exploitation has led many people from Appalachia to distrust outsiders, particularly healthcare providers (McMillan *et al.*, 2007). As our providers with cultural understanding suggested, one approach to bridge this distrust is to utilize Community Health Care Workers (CHWs). CHWs help to develop connections with patients in their own communities, thereby facilitating open communication about their needs for care (Laderman and Mate, 2016). A recent study using CHWs to coach and support diabetes education for adult participants from 26 counties in Appalachian Kentucky showed a significant decrease in HbA_1c_ and a significant increase in feelings of empowerment, diabetes knowledge, and foot and shoe inspection (Feltner *et al.*, 2017). The authors noted that with the shortage of certified diabetes educators and the intense need for diabetes education in rural Appalachia, the use of CHWs allowed for greater cultural competency in reaching people with diabetes in this region (Feltner *et al.*, 2017). Thus, if we can approach Appalachian Ohio as an area including diverse patients in severe medical and psychological need, then providers should be encouraged to adapt culturally competent attitudes and professional skills, which may instill hope about diabetes diagnosis, treatment, and outcomes for both patients and providers in this region.

Appalachia also shares many characteristics of ‘developing’ or ‘low and middle-income’ communities throughout the world where there is a high prevalence of diabetes, a shortage of physicians, and limited economic and social resources that limit access to care. In these communities, *task-shifting*, which includes utilizing paramedical workers instead of physicians to address basic provisions of diabetes care in communities with a shortage of physicians, is often recommended (Misra *et al.*, 2019). Other innovative suggestions for these communities include the use of mHealth techniques such as mobile phones for screening and delivery of diabetes care as well as the use of culturally and linguistically tailored health education interventions (Misra *et al.*, 2019). A recent study conducted in two rural villages in India demonstrated the feasibility of using non-physician health workers (NPHWs) and mHealth to detect and manage diabetes and blood pressure (Dandge *et al.*, 2019). After 24 months of the intervention, 34% achieved target blood glucose and 54% participants achieved target blood pressure (Dandge *et al.*, 2019). These interventions hold promise for addressing the often-cited tenacious socioeconomic, psychosocial, and medical obstacles to diabetes care in Appalachia. If we consider Appalachia as the ‘Invisible Outsider’ (Protivnak *et al.*, 2017) in American life, then we can view it as a unique culture that may benefit from approaches that have been successful in other developing communities worldwide. From this perspective, we may be able instill hope about diabetes diagnosis, treatment, and outcomes for both patients and all providers in this region.

Finally, providers described feeling tired, frustrated, and overwhelmed trying to treat diabetes amidst perceived patients’ fatalism and helplessness, multiple psychosocial issues, and social determinants of health. In a recent study exploring the emotional experiences and attitudes of diabetes healthcare providers, Craven *et al.* (2019) posit the notion of a dynamic relationship between patients’ self-care and providers’ negative emotional experiences whereby less patient engagement produces providers’ negative emotions thereby contribute to even less patient engagement (Craven *et al.*, 2019). Dynamic considerations also were supported in our study with the realization of how one social determinant of health leads to another. Thus, it appears that clinical practice in Appalachia may benefit from thinking about diabetes treatment in a less linear fashion and attempting to understand and address the dynamic interactions among patient fatalism, patient and provider feelings of helplessness, social determinants of health, and the need for providers’ cultural understanding.

Study limitations include homogeneity of the study sample with regards to race/ethnicity, small sample size, participant self-selection, and self-reported data. The predominantly white study sample is reflective of the racial and ethnic distribution in southeastern Ohio (95.0%; Race and Ethnicity in Ohio, 2018; The Appalachian Region, 2020b). The small sample size and participant self-selection also limit the generalizability of the findings. Further, the majority of the participating providers were from Athens County where the medical school is located. Athens County is not a health professional shortage area for primary care providers, which is why the research team was able to interview so many providers. Importantly, the team made sure to interview providers from each county, and for some counties they interviewed the only provider treating patients. As is well known, self-reported data are vulnerable to social desirability bias. Providers with more perceived barriers to diabetes care may have been more willing to participate in the study. Additionally, research with people with diabetes is needed to confirm or contradict the findings from the providers. We conducted a qualitative study with six focus groups (*n* = 36) and nine interviews for a total sample size of 45 participants with diabetes. Currently, we are analyzing the data to identify themes that occurred multiple times, both within and across, the focus groups and interviews. Preliminary findings revealed that people with diabetes identified numerous social determinants of health (e.g., lack of access to care, financial insecurity, limited or no insurance, lack of social support, food insecurity, food deserts) and mental health as significant barriers to their diabetes care (Ruhil *et al.*, 2017). Further, participants perceived a lack of support as well as lack of understanding about Appalachian culture by their providers (Ruhil *et al.*, 2017). The final qualitative analysis is forthcoming. Lastly, the findings from this study, as with all qualitative research, are exploratory and should be considered hypotheses. Future research with a larger, more heterogeneous sample should involve the collection of mixed method data from matched providers and their patients with diabetes to assess objective measures of diabetes self-care behaviors, Hemoglobin A_1c_, psychosocial issues, and social determinants of health.

In conclusion, recognition and understanding of providers’ challenges is an important first step in developing culturally appropriate interventions for people with diabetes in Appalachian Ohio. Culturally appropriate interventions when delivered with community support have been shown to improve diabetes self-care behaviors in other cultural groups, including Hispanic adults with diabetes (Babamoto *et al.*, 2009; Brown *et al.*, 2002; Ramirez and Wu, 2017), African American women with type 2 diabetes (Keyserling *et al.*, 2000; Mayer-Davis *et al.*, 2004), and Asian Americans with type 2 diabetes (Islam *et al.*, 2013; Kim *et al.*, 2015) in the US. Further, studies on ‘low and middle-income’ communities in other countries model how mHealth and NPHWs may offer innovative ways to address diabetes healthcare challenges. Finally, considering the uniqueness of Appalachian culture, interventions tailored to the history, language, and beliefs of this region and its views, like diabetes fatalism, may be more effective than standard diabetes self-management education and support. A brief intervention designed to teach providers cultural competency and cultural humility about Appalachia is needed.
